# Labile organic carbon regulates phosphorus release from eroded soil transported into anaerobic coastal systems

**DOI:** 10.1007/s13280-014-0620-x

**Published:** 2015-02-15

**Authors:** Jouni Lehtoranta, Petri Ekholm, Stella Wahlström, Petra Tallberg, Risto Uusitalo

**Affiliations:** 1 Finnish Environment Institute (SYKE), P.O. Box 140, 00251 Helsinki, Finland; 2 Aalto University, P.O. Box 11000, 00076 Aalto, Espoo, Finland; 3 Department of Environmental Sciences, University of Helsinki, P.O. Box 65, 00014 University of Helsinki, Finland; 4 Natural Resources Institute Finland, 31600 Jokioinen, Finland

**Keywords:** Coastal waters, Agriculture, Sediment, Eutrophication, Fe oxides, Redox reactions

## Abstract

Coastal eutrophication is expected to increase due to expanding and intensifying agriculture which causes a large amount of soil-associated P to be transported into aquatic systems. We performed anaerobic long-term incubations on field soil to mimic the conditions that eroded soil encounters in brackish sediments. The release of P from soil increased with the amount of labile organic C (acetate) addition and decreased with the soil/solution ratio. We deduce that in less-productive brackish systems, microbial Fe reduction allows for the maintenance of the coupled cycling of Fe and P and restricts the amount of P entering the oxic water. In more eutrophic systems, the formation of Fe sulfides as a result of SO_4_ reduction inactivates Fe, and leads to a higher release of P, thus generating an adverse feedback effect. The dependence of the fate of soil-bound Fe and P on the trophic status of the receiving water should be recognized in eutrophication management.

## Introduction

Agricultural phosphorus (P) losses cause eutrophication and hypoxia in coastal waters (Conley et al. 2011). Eutrophication abatement policies tend to rely on total P as the indicator for P loading, and thus give an equal weighting to all P forms, independent of their physical form and bioavailability (see Jansson et al. 2012). Phosphorus is transported from arable land to waters in two major forms: (1) dissolved, readily bioavailable P and (2) soil-bound P that has to be released into a dissolved form before incorporation by biota. Soil-bound P dominates especially in runoff from fine grained soils lacking perennial vegetation (Rekolainen et al. 2006; Ulén et al. 2010).

Erosion control is a widely used P-reduction measure, but it is not clear whether it reduces the losses of bioavailable P. First, tillage practices that aim to prevent soil loss may increase the losses of dissolved P (Uusitalo et al. 2007; Puustinen et al. 2010; Ulén et al. 2010; Daloğlu et al. 2012). Second, the bioavailability of soil-bound P involves complex biogeochemical processes, the outcome of which may be site specific (Ekholm and Lehtoranta 2012). To design efficient agri-environmental measures, the eutrophying effect of soil-bound P should be known. This knowledge is particularly important as agricultural production is expected to expand and intensify (Tilman et al. 2002), and the changing climate may further increase erosion (Puustinen et al. 2007).

The bioavailability of soil-bound P has been estimated by algal assays (DePinto et al. 1981; Ekholm and Krogerus 2003), anion exchange resins (e.g. Uusitalo and Ekholm 2003), and iron (Fe) oxide-impregnated filter papers (Ekholm and Yli-Halla 1992; Sharpley 1993). In general, about 20–30 % of the soil-bound P in agricultural runoff has been found to be bioavailable using the above methods (DePinto et al. 1981; Rekolainen et al. 2006), which, in effect, estimate the P that can be desorbed from eroded soil particles on their way from the field, through rivers to lakes and coastal waters. In other words, the methods mimic the effect of the changing soil/solution ratio (Hartikainen et al. 2010). Sooner or later, the soil particles will end up in sediments, where they will encounter anaerobic conditions. Of the anaerobic mineralization processes, sulfate (SO_4_) reduction may result in a massive P release, because its end-products, sulfides (e.g., HS^−^, H_2_S), inactivate the major P-sorption component, Fe oxides (Roden and Edmonds 1997). Extraction with buffered sodium dithionite (Na_2_S_2_O_4_) has been considered as mimicking the P release mediated by sulfides, and it has captured as much as 60 % of the total P in Finnish clayey soils (Uusitalo and Turtola 2003). However, it is not known whether dithionite extraction describes the P release which occurs through anaerobic microbial processes that couple the cycles of carbon (C), Fe, sulfur (S), and P in sediments (Burgin et al. 2011). Using the theoretical basis of coupled biogeochemical cycles, Lehtoranta et al. (2008, 2009) have proposed that the release of P from sediments is related to regional variations in Fe and SO_4_ reduction and that this variation stems from the varying supply of organic C.

Here, we hypothesize that the bioavailability of soil-bound P in marine and brackish water is related to the supply of organic C. We carried out anaerobic soil incubations with varying organic C amendments and soil/solution ratios to study the behavior of soil-bound P encountering anoxic brackish conditions. The aim was to use the results for discussion of how the trophic status of the coastal system may control the behavior of soil matter P settled at the bottom. The study highlights that the extent of sedimentary microbial processes needs to be taken into account when planning water protection measures for the brackish systems such as the Baltic Sea.

## Materials and methods

### Soil and its analysis

The surface (0–20 cm) soil used in the incubations has been taken from a cultivated field (Tammela, southern Finland, 60°49.228′N; 23°44.47′E). It is a silty clay by texture and has a moderate P status according to Finnish and high P status according to other agronomic indices (Table 1). To determine “pH-sensitive” and “alkali-extractable” pools of P, the soil (400 mg) was extracted with a nutrient medium (40 ml, see below) adjusted by Tris buffer (see below) at pH 9, and with 0.1 M NaOH (40 ml). Both extractions were performed repeatedly five times (1 h each) with centrifugation between the extractions and analysis of the supernatant. The soil was also extracted with buffered dithionite according to Uusitalo and Turtola (2003). The method was originally used to characterize P in runoff that contains eroded soil, and for a higher confidence of the estimate when applied to soil analysis, the extraction was done using a dilution series with soil concentrations of 0.62, 0.98, 1.50, 1.79, 2.62, and 3.00 g in a liter of deionized water. One milliliter of 0.298 M NaHCO_3_ (as a pH buffer) and 1 ml of 0.574 M Na_2_S_2_O_4_ (as the reducing agent) were added to 40 ml of soil–water suspension, the samples were immediately capped, shaken for 15 min, filtered (0.2-µm Whatman/Nuclepore polycarbonate filters), and analyzed for total dissolved P (filtered samples digested in an autoclave with peroxodisulfate-sulfuric acid) using a LaChat QuickChem 8000 flow injection analyzer (general detection limit 3 µg L^−1^, and for BD extraction 23 µg L^−1^) employing molybdate colorimetry and for dissolved Fe with an inductively coupled plasma-optical emission spectrometer (ICP-OES, diluted in 1/1 with 6 M HCl to reduce reprecipitation of Fe in aerobic environment). The digestion efficiency for BD extraction for Fe precipitate treated with P addition was 72 % (Uusitalo and Turtola 2003). A similar extraction by water was also done (soil concentrations 0.60, 0.95, 1.70, 2.18, 2.63, and 3.25 g l^−1^). The results reported in this paper refer to the difference between dithionite-extractable P and water-extractable P at each soil/solution ratio.

**Table 1:** Characteristics of the soil used in the incubations

Variable	Extraction	Unit	Value
Phosphorus	0.5 M ammonium acetate-acetic acid, pH 4.65^a^	mg l^−1^	15.0
	Water	mg g^−1^	0.022–0.030^e^
	Repeated nutrient medium, pH 9	mg g^−1^	0.050
	Olsen-P	mg kg^−1^	69.0
	Mehlich-3 P	mg l^−1^	95.0
	Anion exchange resin^b^	mg g^−1^	0.12
	Buffered dithionite	mg g^−1^	0.13–0.21^e^
	Repeated 0.1 M NaOH	mg g^−1^	0.57
	Total^c^	mg g^−1^	1.47
Iron	Buffered dithionite	mg g^−1^	1.7
	Oxalate^d^	mg g^−1^	9.7
Aluminum	Oxalate	mg g^−1^	2.5
Ca	0.5 M ammonium acetate-acetic acid, pH 4.65^1^	mg l^−1^	3030
K	0.5 M ammonium acetate-acetic acid, pH 4.65^1^	mg l^−1^	370
Mg	0.5 M ammonium acetate-acetic acid, pH 4.65^1^	mg l^−1^	1060
C		% DW	2.3
N		% DW	0.19
pH			6.6

^a^Finnish soil test analysis (Vuorinen and Mäkitie 1955)

^b^Analysis based on Sibbesen (1977)

^c^Analysis based on Bowman (1988)

^d^Analysis based on Schwertmann (1964) using ICP-OES

^e^Minimum and maximum of the extractions with six soil/solution ratios

### Anaerobic incubations

**Fig. 1:**
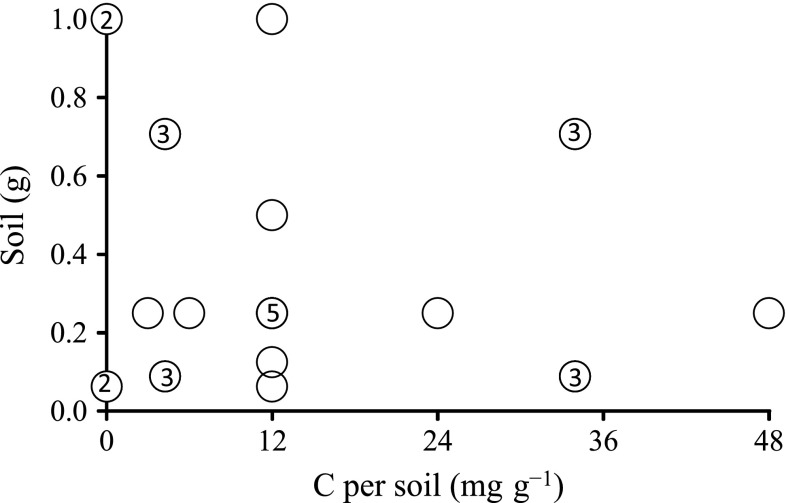
Experimental design following a response surface methodology. The *circles* denote the experimental units, and the *numbers* inside the *circles* refer to replicates ranging from 2 to 5. The number is missing when there were no replicates; in *Incubation 2*, two replicates were used instead of three

To estimate the mutual effects of organic C and soil/solution ratio on anaerobic P release, various amounts of soil were incubated by adding organic C in the form of acetate as an electron donor. The absence of O_2_ and NO_3_ guaranteed that there were only three electron acceptors present: Fe and Mn oxides provided by the soil and SO_4_ by the brackish medium. Note that the experimental set-up did not allow for the renewal of the Fe oxide pool through endogenous oxidation processes by O_2_ and NO_3_, i.e., the ‘ferrous wheel’ was inactivated. The experimental design followed a response surface methodology with a central composite design, i.e., a first-order design augmented by additional center and axial points, with soil and organic C acting as variables. Five replicates were placed at the central point (0.25 g soil, 3 mg C, Fig. 1).

Two incubations were performed. In *Incubation 1*, air-dried soil was weighed (levels: 0.062, 0.088, 0.125, 0.250, 0.500, 0.707, and 1.0 g) into 100-ml glass bottles developed for anaerobic incubations. In addition to this, there were three control units containing test solution only. The bottles were filled with 80 ml of filtered water (0.2 µm polypropylene membranes) from the coastal Gulf of Finland (Karpinselkä, Helsinki, about 5 practical salinity units; UNESCO, 1981) containing 310 mg l^−1^ of SO_4_. Sodium acetate (CH_3_COONa) was added at levels: 0, 0.375, 0.75, 1.5, 3, 6, 12, and 24 mg C. The solutions’ pH was buffered to 8.1 by diluting 1.51 g of the Tris buffer (tris(hydroxymethyle)aminomethane) Sigma Trizma base with 25 ml of distilled water. Each bottle was inoculated with 10 µl of surface (0–1 cm) sediment from the Gulf of Finland (Vanhankaupunginlahti Bay, Helsinki) and bubbled with N_2_ gas. To simulate the cold sedimentary environment of the Gulf of Finland and the long-term effect of the geochemical processes, the bottles were incubated at 5°C close to temperature of bottom waters in the dark for 861 days. This was anticipated as enough time to demonstrate the outcome of the long-term geochemical processes on P release from soil settled in an anoxic environment. On days 738 and 861, the solution concentrations of Al, Ca, Fe, K, Mg, Mn, Na, P, and S were measured using ICP-OES (Thermo iCAP6000) after filtration (WWR International 25-mm Syringe Filter, polypropylene membrane with 0.2-μm pore size). SO_4_ was determined by ion chromatography, H_2_S colorimetrically with a prepared cuvette test (Hach Lange LCK 653), and pH potentiometrically.

*Incubation 2* was similar to *Incubation 1*, apart from the test solution, the duration, and the temperature of the experiment. Moreover, C was added also during the incubation (on days 141 and 196) to establish the effect of labile C on a stabilized system. The test solution consisted of a P-free nutrient medium 5 % Z8 (Kotai 1972) with a salt component (about 6 practical salinity units, Ekholm et al. 2009). To study the effect of a higher SO_4_ concentration, K_2_SO_4_ was added to give an initial concentration of 2.4 g l^−1^ SO_4_, corresponding to that of sea. Moreover, NH_4_ was added to ensure sufficient N for bacterial activity to yield a concentration of 2 mg l^−1^ N, a level found in pore waters of the sediments in the Gulf of Finland (Lehtoranta and Pitkänen 2003). The incubation took place on a shaking table at 10°C in the dark for 308 days. At the end of the incubation, pH, dissolved reactive P (molybdate blue colorimetry), and dissolved Fe were analyzed.

### Statistical analysis

A multivariate regression analysis was performed using SAS for Windows (9.3) to investigate the dependences of P release (*P*, mg g^−1^) on soil (*soil*, g) and C (*C*, mg, Eq. 1). The regression model included two terms. The first term was assumed to account for P desorption and was an inverse exponential function of soil, i.e. the more soil there was in a unit, the lower the amount of P released per gram of soil. The form followed the experimental results of e.g., Yli-Halla and Hartikainen (1996) and Yli-Halla et al. (1995). The second term was assumed to represent the effect of Fe reduction (either microbiological or mediated by H_2_S) on the P release. Here, the mineralization of organic C was assumed to follow first-order reaction kinetics. The assumption was justified by the use of a single and readily available C source (acetate), although the organic matter in soil, exhibiting a more complex degradation (Vähätalo et al. 2010), may also have been mineralized in the experiment.

1 $$ P = P_{\text{soilmax}} \cdot e^{{ - k_{1} \cdot {\text{soil}}}} + P_{C\hbox{max} } \cdot (1 - e^{{ - k_{2} \cdot C}} ). $$

The coefficients *P*_soilmax_, *P*_*C*max_, *k*_1_ and *k*_2_ were numerically approximated with PROC NLIN in SAS. The model’s goodness of fit was evaluated with the Nash–Sutcliffe efficiency index *E*_f_ (Nash and Sutcliffe 1970):

2 $$ E_{\text{f}} = 1 - \frac{{\mathop \sum \nolimits_{i = 1}^{n} (\widehat{Y}_{i} - Y_{i} )^{2} }}{{\mathop \sum \nolimits_{i = 1}^{n} (Y_{i} - \overline{Y} )^{2} }}, $$

where $\widehat{Y}_{i}$ is the modeled value, and $Y_{i}$ is the observed value, $\overline{Y}$ is the average of the observed values, and *n* is the number of observations. The index ranges from −∞ to 1, with a value of 0 indicating that the predictions are as accurate as the mean of the observations and a value of 1 indicating a perfect match.

## Results

### Soil P and Fe

The P extracted by buffered dithionite ranged from 0.13 to 0.21 mg g^−1^ (Table 1), and the extracted P increased with the decreasing soil/solution ratio of the extraction (data not shown). The upper boundary accounted for 17 % of the total P in soil and was about twice the concentrations of anion exchange resin-extractable P and Mechlich-3 P and tenfold the concentration of water-extractable P (Table 1). A higher amount of P was removed by the repeated NaOH extraction (0.57 mg g^−1^), while the repeated extraction by nutrient medium at pH 9 gave only 0.05 mg g^−1^ of P. Of the soil Fe, 1.7 and 9.7 mg g^−1^, were extracted by dithionite and oxalate, respectively (Table 1). Of the oxalate-extractable metals, the mass of Fe was about four times that of aluminum (Al), the molar ratio being about two (175 mmol kg^−1^ Fe vs. 95 mmol kg^−1^ Al).

### Mineralization processes

**Fig. 2:**
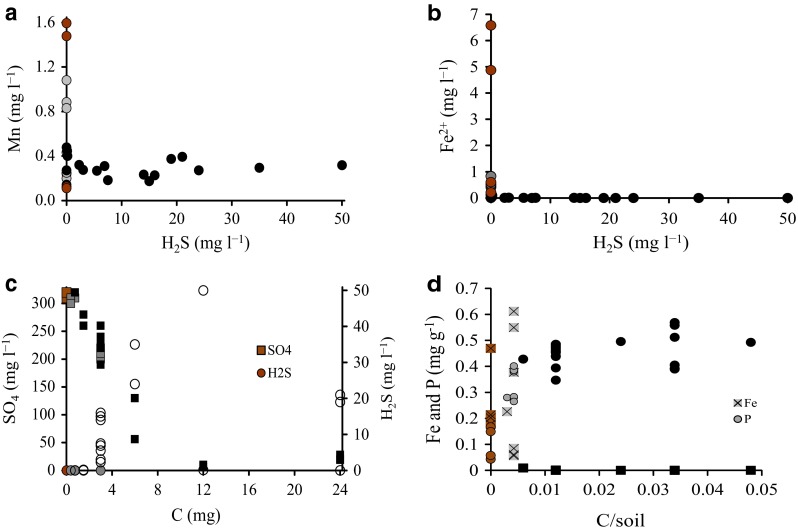
Relationship between **a** the solution concentrations of manganese (Mn) and hydrogen sulfide (H_2_S); **b** reduced iron (Fe^2+^) and H_2_S; **c** sulfate (SO_4_) and H_2_S and addition of carbon (C); and **d** C/soil ratio and iron (*squares*) and phosphorus (*circles*) released from soil to solution. *Brown symbols* represent the units without addition of C, *gray symbols* the units with C/soil ratios <6, and *black symbols* the units with C/soil ratios ≥6

**Fig. 3:**
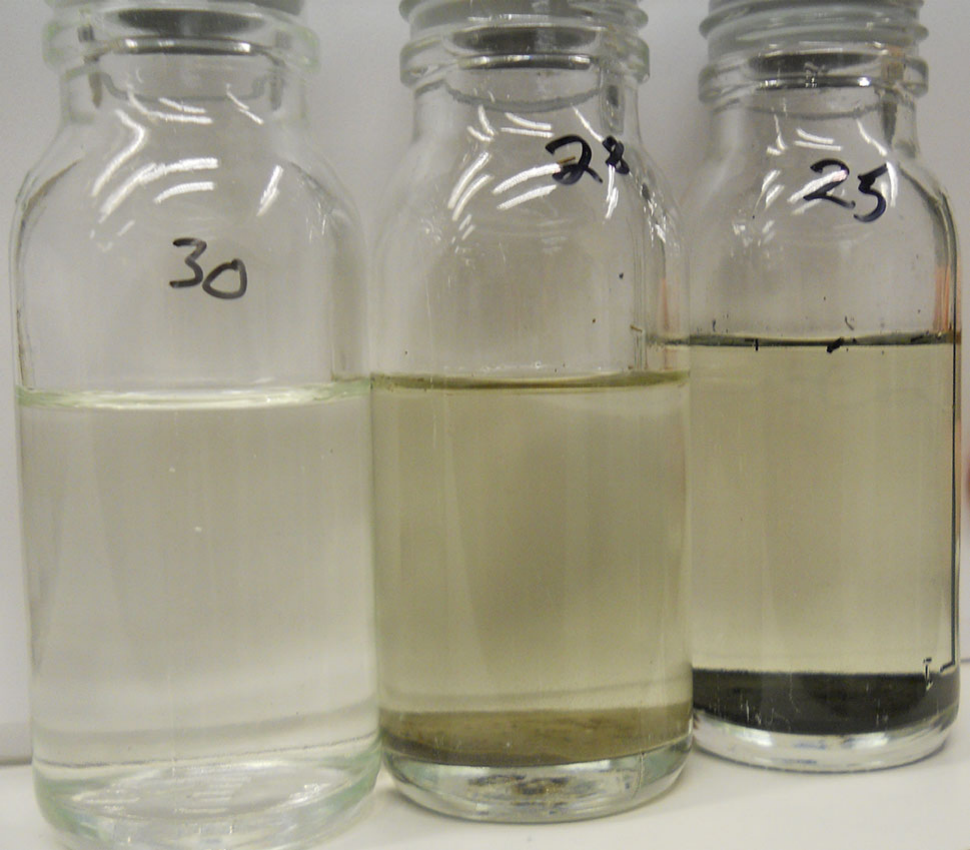
The water of the Gulf of Finland without soil remained clear (*left*), and the units without addition of C (here 1.0 g soil) kept their brownish color (*middle*), but units with high C/soil ratio (here 24 mg C and 0.71 g soil) turned black (*right*) in the anaerobic incubation. Photo by Jouni Lehtoranta

Indices of anaerobic mineralization processes were detected in all the experimental units. Even in the units without acetate addition, Mn and Fe accumulated in the solution, suggesting that the organic C present in the soil was able to launch Mn and Fe reduction (brown circles in Fig. 2a, b). Dissolved Fe was only detected in the units with C/soil ratios (mg g^−1^) of less than 6 (brown and gray circles in Fig. 2b). Higher C/soil ratios (black marks in Fig. 2) appeared to enable SO_4_ reduction, as suggested by a strong decrease in SO_4_ concentration, accumulation of H_2_S (Fig. 2c), and smell. The end-products of Fe and SO_4_ reduction, dissolved Fe and H_2_S, were not detected simultaneously in solution, suggesting efficient coprecipitation (Fig. 2b). The black color of the units with high C/soil ratios provided further evidence of the presence of Fe sulfides. A simultaneous release of dissolved Fe and P from soil was observed in the units with low C/soil ratio (Fig. 2d). A further increase in the C/soil ratio resulted in negligible concentration of Fe. The units with low (or no) C additions maintained their initial brownish color throughout the experiment (Fig. 3) and showed a low consumption of SO_4_ and no accumulation of H_2_S. Similar visible processes were observed in *Incubation 2* until extra C was added and all units turned black.

**Fig. 4:**
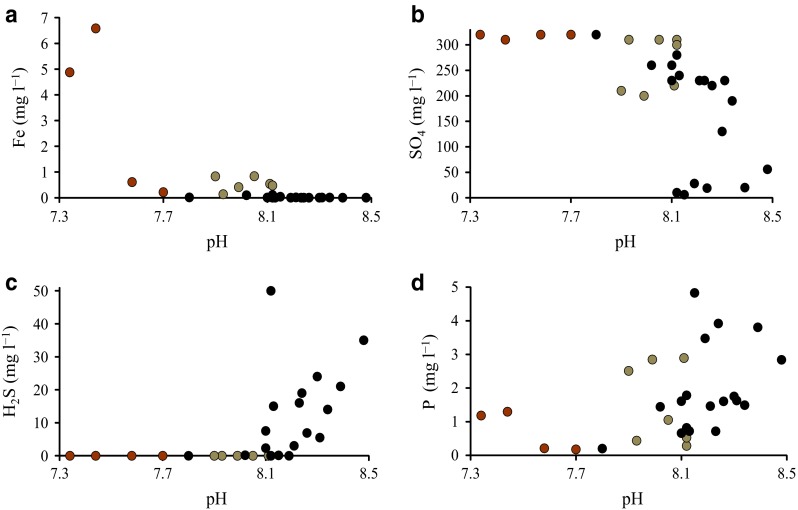
Relationship between **a** pH and solution concentrations of iron (Fe); **b** pH and sulfate (SO_4_); **c** pH and hydrogen sulfide (H_2_S); and **d** pH and phosphorus (P). The *brown* *symbols* denote units without addition of organic carbon (C); the *gray* the units with C/soil ratios <6; and *black* *symbols* the units with C/soil ratios ≥6

The highest pH values were measured in the units with efficient SO_4_ reduction and high H_2_S concentrations (Fig. 4a–c). Although the highest P release was found in the units with the highest pH, the increase in pH was hardly the principal cause for P release (Fig. 4d), as described below.

### Effect of C/soil ratio on the release of P

**Fig. 5:**
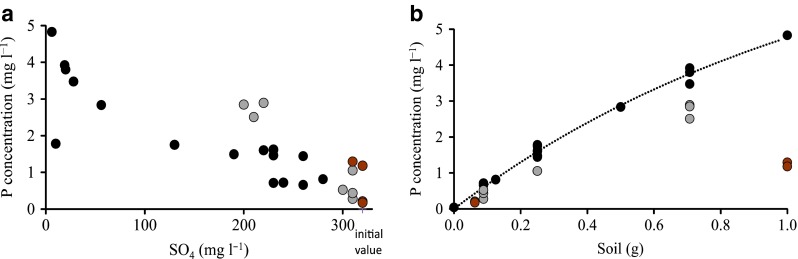
Relationship between **a** the solution concentration of phosphorus (P) and sulfate (SO_4_). Low concentrations of P were generally associated with high concentrations of SO_4_. **b** The relationship between P concentration in solution and amount of soil (grams per 80 ml of suspension). The *brown* *symbols* denote units without addition of C, *gray* *symbols* the units with C/soil ratios <6, and *black symbols* the units with C/soil ratios ≥6

The release of P correlated inversely with SO_4_ concentration (Fig. 5a), i.e., positively with SO_4_ consumption. This pattern can be explained by the collapse of the P binding ability of Fe by sulfides formed as a result of SO_4_ reduction. When the C/soil ratio exceeded 6, the solution P concentration was a logarithmic function of soil concentration, but at lower C/soil ratios, the P concentrations were lower and also predicted by C concentration (Fig. 5b). For example, the units with 1 g of soil, but no addition of C had an average P concentration of 1.25 mg l^−1^ (*n* = 2), whereas the unit with the same soil amount but 12 mg C had a P concentration of 4.8 mg l^−1^. No corresponding change was found in exchangeable cations during the incubation (Table 2).

**Table 2:** Variation (min–max) of exchangeable cations in test solution and suspensions (with and without addition of carbon) at the end of the incubation. DL = the lower detection limit

Experimental units	*n*	Al (mg l^−1^)	Ca (mg l^−1^)	K (mg l^−1^)	Mg (mg l^−1^)	Na (mg l^−1^)
Test solution	3	<DL	70–71	56	172–174	1340–1360
No added carbon	4	<DL–0.06	71–90	46–56	170–175	1320–1350
Added carbon	25	<DL–0.11	69–83	39–55	151–172	1320–1500

**Fig. 6:**
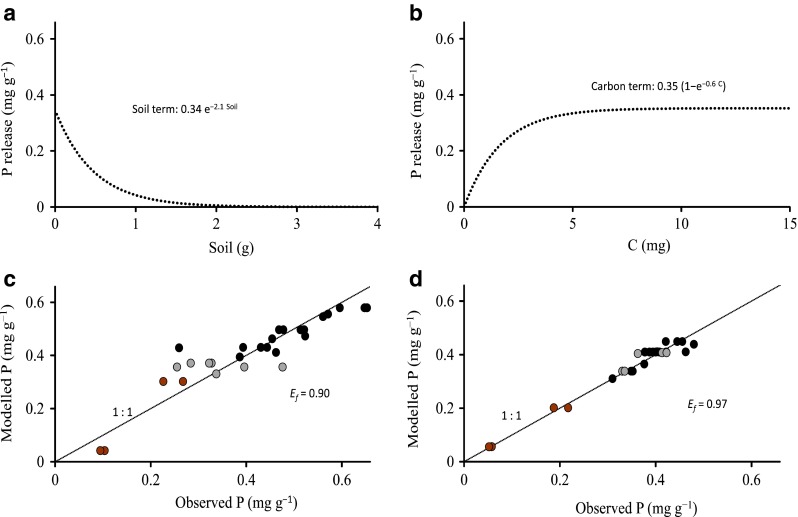
**a** Effect of ‘soil term’ on the P release in *Incubation 1* (861 days). **b** Effect of organic ‘carbon term’ on the P release. **c** Observed and modeled P releases in *Incubation 1*. **d** Observed and modeled P releases in *Incubation 2* when the extra addition of organic C increased the C/soil ratio over 6 in all units. *Brown* *symbols* represent the units without addition of C; *gray symbols* the units with C/soil ratios <6; and *black* *symbols* the units with C/soil ratios >6. In **d**, the *colors* denote the C/soil ratio of the starting point of the experiment without the extra addition and the actual C/soil ratio is >6 for all units. *E*_f_ is the model efficiency index

The regression model predicted the P release well, with the *E*_*f*_ values ranging from 0.78 to 0.97 (Table 3; Fig. 6c). The theoretical maximum P releases, determined by letting *soil* approach 0 and *C* infinity, were 0.70 mg g^−1^ in *Incubation 1* and 0.47 in *Incubation 2* (Table 3 showing the results for *Incubation 2*). Figure 6 depicts the effects of soil and C terms on the P release. The soil term appeared to overestimate desorption with low soil contents; the soil term indicated a P release of about 0.3 mg g^−1^ at low soil contents, which is a much higher value than that obtained by anion exchange resin (0.12 mg g^−1^) and repetitive extraction by nutrient medium (pH 9, 0.057 mg g^−1^). The C term suggested that as much as 97 % of the P mobilized by C was already released at *C* = 6 mg.

**Table 3:** Regression equations for P release (*P*) in *Incubation 1*. *P*_max_ is the maximum P release, *E*_f_ is the model efficiency index. Soil is given in grams and *C* in milligrams

Duration	Phosphorus release (mg g^−1^), soil ≠ 0	*P*_max_	*E*_f_
738	*P* = 0.27·e^*−*3.2·soil^ + 0.33·(1 − e^*−*1.5·*C*^)	0.61	0.90
861	*P* = 0.34·e^*−*2.1·soil^ + 0.35·(1 − e^*−*0.6·*C*^)	0.70	0.83

In *Incubation 2,* the response of an extra C addition was tested after 141 and 196 days. The extra C increased the P release only in the units with a low C/soil ratio. The extra C addition improved the fit of the model by increasing the dependence of P release from the amount of soil but not increasing the estimate for maximum P release (Fig. 6d). Therefore, the extra dose of organic C led to the release of P that could be modeled with the soil term alone. Sulfate itself seemed to have a small effect on P release: the high initial SO_4_ concentration (2400 mg l^−1^) in *Incubation 1* did not increase the P release from the soil in comparison with the lower initial value 310 mg l^−1^ of *Incubation 2*.

**Fig. 7:**
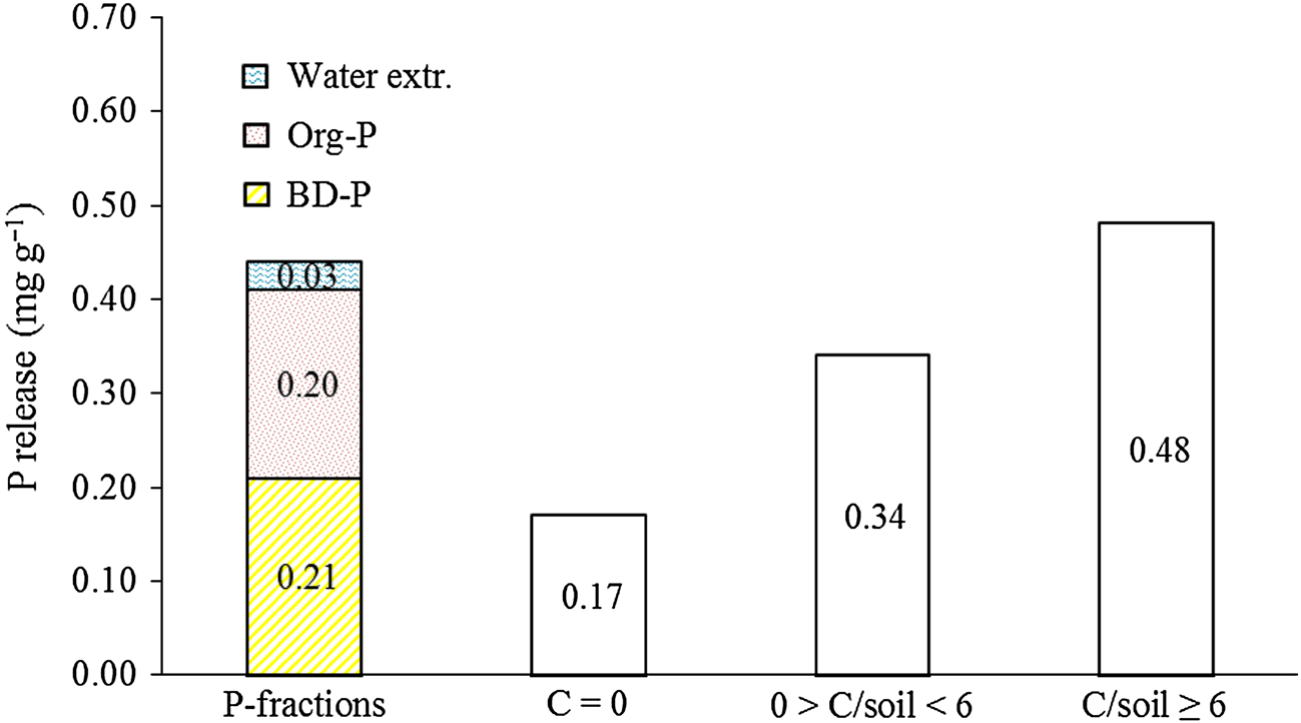
Phosphorus fractions compared with average P released in different C/soil ratios (*C* = 0 refers to no C addition) showing the significance of the addition of C on release of P. Phosphorus fractions consist of the P extracted with water and buffered dithionite (BD), and the estimated organic-P

The average P release in units with C/soil ≥6 was 0.48 mg g^−1^, more than twice the amount extracted with buffered dithionite (Fig. 7). The average P release was close to the sum of (1) P extracted by dithionite; (2) P extracted by water (soil/solution 1/100); and (3) the estimated amount of organic P, the latter approximated from the C concentration of the soil. The maximum P release (0.65 mg g^−1^) was about three times the amount extracted by dithionite and 44 % of the total P in the soil sample.

## Discussion

### Carbon and progressing microbial reduction

In our bottle test, the redox-states, pH, and the concentrations of Mn, Fe, SO_4_, and H_2_S prevailing at the end of incubations were typical of those found in the sediments of brackish systems, such as the open and coastal Baltic Sea (see e.g., Lehtoranta and Heiskanen 2003; Reed et al. 2011). In sediments, most of the organic C consists of compounds which are not readily available for mineralization. It is likely that the mineralization is controlled by the ability of the sediment to produce labile C compounds (e.g., by fermentation) rather than by the concentration of C itself. In our experiment, acetate was used to simulate a system that was able to produce labile C for microbial mineralization using Mn, Fe, and SO_4_ as electron acceptors. By manipulating the supply of labile organic C to brackish soil suspensions, we initiated progressing redox evolution, analogous to that encountered by the eroded soil when it settles on anoxic sediments with different abilities to produce labile C.

When no acetate was added, the organic C present in the soil was able to produce Mn and Fe in the solution, but SO_4_ remained at the initial level. Low additions of acetate maintained the accumulation of Mn and Fe and resulted in a minor decrease in SO_4_ (Fig. 2a–c). Thus, a low C level could trigger the microbial reductions of Mn and Fe, but the system did not proceed into SO_4_ reduction with such an efficiency that it would have markedly decreased the level of SO_4_, even though *Incubation 1* took more than 2 years. However, SO_4_ reduction was activated with higher additions of acetate. At moderate C levels, the concentration of SO_4_ decreased, but no H_2_S was detected. The lack of H_2_S was possibly due to coprecipitation of sulfides and Fe^2+^ in the solution (Chapelle et al. 2009), as suggested by the fact that the concentration of dissolved Fe was lower in these units.

A high C addition was required to exhaust the reserves of reactive Mn and Fe oxides in the soil, giving room for the dominance of SO_4_ reduction. When the C/soil ratio was at least 6, soils in all units turned black, as a result of the transformation of Fe(III)oxides into black Fe sulfides. Furthermore, the concentration of dissolved Fe became nondetectable and H_2_S accumulated into the water. The presence of H_2_S indicates that even the abiotically reducible Fe oxides may have precipitated as Fe sulfides. The microbial and chemical processes in the experiment produce many simultaneous reactions with an opposite effect on pH (Soetaert et al. 2007), but in general, the pH was the lowest when dissolved Fe was present (low C additions) and the highest in units with presence of H_2_S (high C additions, Fig. 4a–d).

### Organic C and P release

Of the elements monitored, P was the only nonredox-dependent element that increased in concentration as a function of organic C (Table 2). However, after exceeding a threshold in C/soil ratio, further C additions caused lower additional P release, and the release could be modeled well with the amount of soil alone (Fig. 6d). The diminishing increase in P release from soil (Fig. 5b) could be due to, for example, P readsorption on Fe(II) hydroxides, Al oxides, and mineral surfaces (Patrick and Khalid 1974; Roden and Edmonds 1997; Gächter and Müller 2003).

When no C was added, the release of P was accompanied by the release of Fe, with the molar Fe/P ratio in solution ranging from 0.7 to 2.8 (calculated from data in Fig. 2d). This ratio indicates that microbial Fe reduction results in an accumulation of both Fe and P, and presupposes that Fe has the capacity to bind most of the released P, when oxic conditions are encountered (Blomqvist et al. 2004). Therefore, the eroded soil may carry enough Fe to capture the P released through this coupling of the Fe and P cycles in the brackish sedimentary systems, but our results indicate that this ability is maintained only in environments low in C.

In the units with C/soil ratio <6, the molar Fe/P ratio ranged from 0.02 to 0.50 and in the units with C/soil ratio ≥6, the ratio was at maximum 0.1 and mostly zero. Thus, organic C had the ability to decouple the cycles of Fe and P, through SO_4_ reduction, by deteriorating the capacity of Fe to retain P. The pattern found follows the theory presented by Lehtoranta et al. (2009). Those authors hypothesized that anoxic oligotrophic brackish and marine sediments may be able to maintain the coupled Fe and P cycling, but if eutrophication proceeds, the ability of Fe to retain P is lost through the efficient reduction of SO_4_. Our results also endorse the previous study by Lehtoranta et al. (2008) which states that the ability of the surface sediment to retain P is related to the difference in the dominance of microbial Fe and SO_4_ reductions in the bottom areas of the Baltic Sea. However, we have only studied the behaviors of Fe, Mn, and P in a clayey soil, and our study gives little information about the fates of Fe and P bound, e.g., to humic matter in SO_4_-rich sedimentary systems. The forms of C, Fe, and P and their respective loadings from rivers to the subbasins of the Baltic Sea vary significantly from one region to another. The recipient sedimentary system may also respond differently to each load type, and the release of P may depend on the binding and the molar ratios of C, Fe, and P in the terrestrial material.

The P release in the units with high C/soil ratio exceeded the amount of P extracted by buffered dithionite that has earlier been used to estimate the mobile P in sulfidic sediments. The difference may be explained by the mineralization of organic P present in significant amounts in the test soil, and also by the duration of the anoxic period—15 min in the dithionite extraction as opposed to over two years in the incubations. During the incubation, microbes and sulfides may be able to liberate P also from the less readily accessible metal oxides (such as P in interiors of Fe oxides). In addition, the organic anions, such as acetate, may have competed with PO_4_ for adsorption sites (on organic anion competition, see, e.g., Froelich 1988), and the increased pH may have promoted P desorption via anion exchange with OH^−^ and through the decreasing positive charge of oxide surfaces (Hingston et al. 1967; Caraco et al. 1989; Hawke et al. 1989; Hartikainen and Yli-Halla 1996). The addition of acetate was likely to elevate pH both directly due to its weak basicity (pKb ≈9.3) and indirectly via SO_4_ and Fe reductions (Ben-Yaakov 1973; Lamers et al. 1998; Soetaert et al. 2007; Boudreau and Canfield 1993).

Our experimental approach may give insight into the mobility of soil-bound P under conditions where P-release is controlled by anaerobic microbial processes, serving as an additional method besides algal assays and chemical extractions. The long incubation may in part reflect realistic dynamics of microbial processes affecting the P release in brackish and marine sediments. In future tests, a shorter incubation time may be compensated by increasing the temperature. However, particulate organic P may behave differently compared to P bound to reactive Fe oxides. Studies from shallow coastal sediments have shown that the efficient reduction of Fe and release of P can be measured within weeks (Jensen et al. 1995; Kristiansen et al. 2002), but that the mineralization of organic P may take years in anoxic sediments (Ahlgren et al. 2006). Therefore, a short incubation time may reveal better the release of Fe bound P than that of organic P.

## Conclusions

Under anoxic conditions, the release of P from soil was controlled by organic C, suggesting that the mobility of P depends on the trophic status of the sedimentary system, i.e., mineralization potential created by labile organic C. Our results propose that erosion control measures affecting the quantity and quality of fluxes of Fe and P may have a complicated outcome when it comes to eutrophication in the receiving water bodies. In an oligotrophic SO_4_-rich system, the soil matter settling at the bottom may carry enough Fe to prevent most of the P entering the surface waters. However, in a eutrophic SO_4_-rich system, the ability of Fe to capture P is lost by the Fe sulfide formation. This highlights that the efficiency of erosion control as a eutrophication abatement measure may to a large extent depend on the characteristics of the receiving aquatic system.
